# Supplementary material for: CRISPR-mediated multiplexed live cell imaging of nonrepetitive genomic loci with one guide RNA per locus
Source: Nat Commun. 2022 Apr 6;13:1871. doi: 10.1038/s41467-022-29343-z (PMC8987088; doi:10.1038/s41467-022-29343-z)
Supplement: Supplementary file 1 — Supplementary Information [file 41467_2022_29343_MOESM1_ESM.pdf]

CRISPR-mediated multiplexed live cell imaging of nonrepetitive genomic loci with one guide RNA per locus

Supplementary Information

Clow *et al*.

Supplementary Table 1. Comparison of Casilio with published CRISPR-based imaging systems for visualizing endogenous unmodified genomic loci.

**Reference in main text**	**Composition**	**Minimal number of gRNAs for non-repeat imaging**	**Number of colors**
Chen 2013[14]	dCas9-EGFP	36	1
Tanenbaum 2014[15]	dCas9:SunTag$_{24x}$:GFP	N.A. (only repeats)	1
Ma 2016[16]	dCas9:MS2/PP7/boxB:FP	N.A. (only repeats)	3[a]
Qin 2017[17]	dCas9:16xMS2:mCherry	4	1
Ma 2018[18]	dCas9:8xMS2/PP7:FP	N.A. (only repeats)	2
Maas 2018[19]	dCas9:MS2/PP7:FP	2[b]	2
Gu 2018[20]	dCas9-EGFP	12	1
Wang 2019[21]	dCas9:gRNA-Cy3	N.A. (only repeats)	2
Mao 2019[22]	dCas9:gRNA:MB(FRET)	3	1
Casilio (this study)	dCas9:PUF:FP	1	3

[a]Using combination of 3 colors, co-imaging of a maximum of 6 loci was demonstrated. However, this relies on color combination. When labeling very close-by loci, deconvolution of color combinations may become a challenge.
[b]More than expected number of spots observed, requiring elaborate filtering algorithm keeping two brightest spots (see supplementary figure 3 of that reference).

Supplementary Table 2. **Listing of Plasmids with links to plasmid maps and Addgene.**

Code	Plasmid Name	Plasmid map on Benchling	Addgene Link
pAC11001	lenti_dCas9	https://benchling.com/s/seq-nwhb6COmj4HyvRDaltcT?m=slm-0GaEau5AEgsDsPVvBi9D	https://addgene.org/183208
pAC1447	pmax-Clover_PUFc	https://benchling.com/s/seq-DjznMYu2SHLIVqh8JjQ3?m=slm-XhF9XSW3mbTfMEgZzO4W	https://addgene.org/73689
pAC11002	pmax-PUF9R_iRFP670	https://benchling.com/s/seq-ymk2raAqiMP1wZEIDtmX?m=slm-XLBZX3AQozvu2RDxWiUi	https://addgene.org/183209
pAC11003	pmax-PUF9R_mRuby2	https://benchling.com/s/seq-05CQGYUdjnefm6nwJrkI?m=slm-meYSbUjvCBrc744J7tSZ	https://addgene.org/183210
pAC11004	pmax-Clover_PUF48107	https://benchling.com/s/seq-fRaBdNkydw9dDeyowhdd?m=slm-rDks2uTpsak4mVz0w3Ml	https://addgene.org/183211
pAC11005	pmax-iRFP670_PUFc	https://benchling.com/s/seq-6tmSjU4WcZpzy03YcpEv?m=slm-eDyvA9z21iWDM1HU8IH1	https://addgene.org/183212
pAC11006	pCR8-sgRNA-15xPBSc	https://benchling.com/s/seq-XFhAw0mDVUoNViBilYLH?m=slm-BkR0AYIqz45RygiksM1X	https://addgene.org/183213
pAC11007	pCR8-sgRNA-15xPBS9R	https://benchling.com/s/seq-mj21Noq9avBms6Ooa31D?m=slm-nQbglQHY0PjDhlr1EVTT	https://addgene.org/183214
pAC11008	pCR8-sgRNA-20xPBS48107	https://benchling.com/s/seq-BMHoDIBHYEfLM9Meyp4p?m=slm-L00jM5Knz4LpNvSFSG6Y	https://addgene.org/183215
pAC11009	pCR8-sgRNA-20xPBSc	https://benchling.com/s/seq-MHL6lsmXICnDbdldVjFD?m=slm-1gbixcNOTO25ngnYOhYo	https://addgene.org/183216
pAC11010	pCR8-sgRNA-25xPBS9R	https://benchling.com/s/seq-fOBZj7IIuZjAoxDEy44I?m=slm-l7OYnvymuAzct16VuTes	https://addgene.org/183217

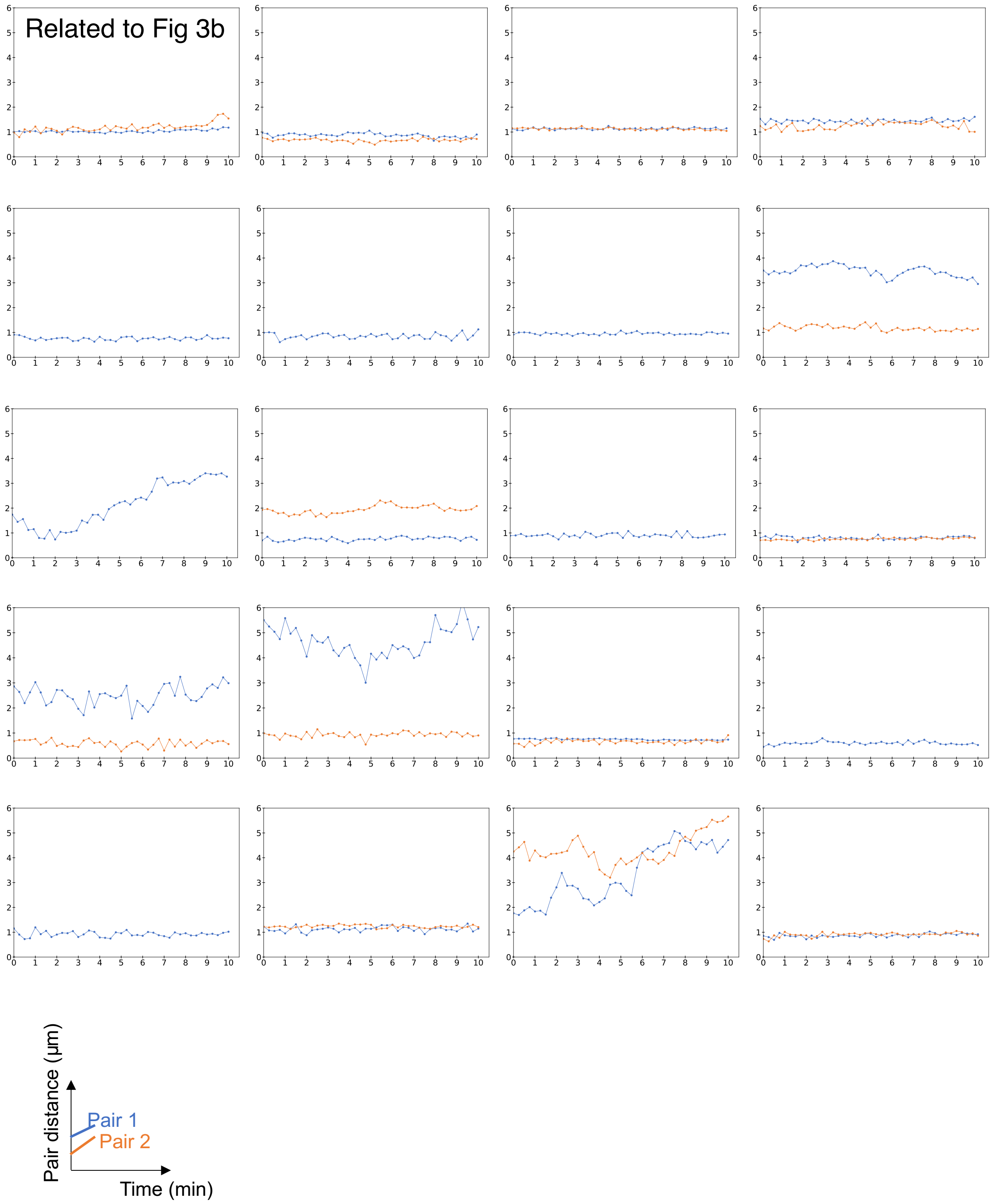

Supplementary Fig. 1. Pairwise 3D distances of Clover and iRFP670 foci labeling the *MASP1*-*BCL6* loop in 20 ARPE-19 nuclei (33 pairs) over time. Source data are provided as a Source Data file.

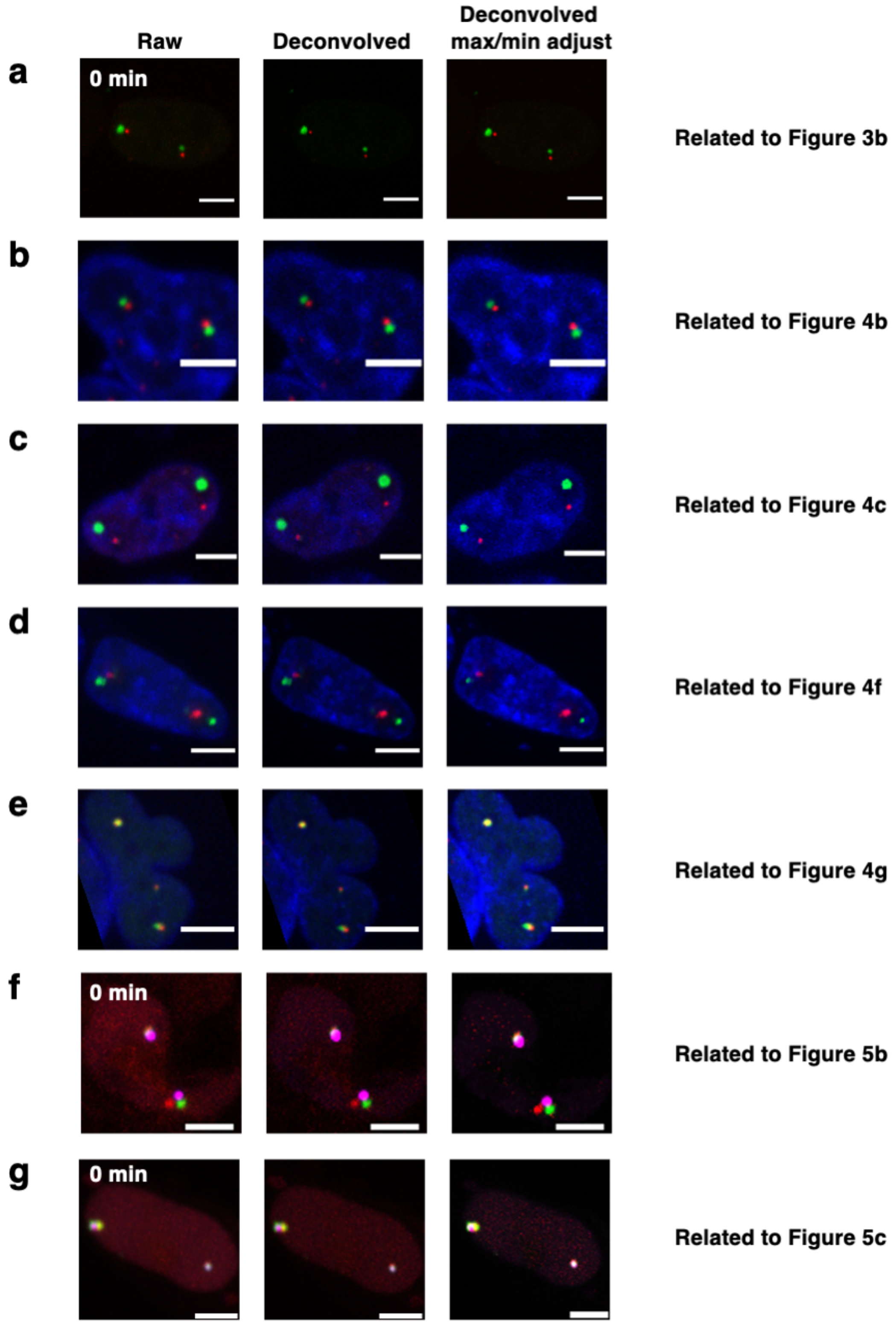

Supplementary Fig. 2. Example images through each step of image processing.

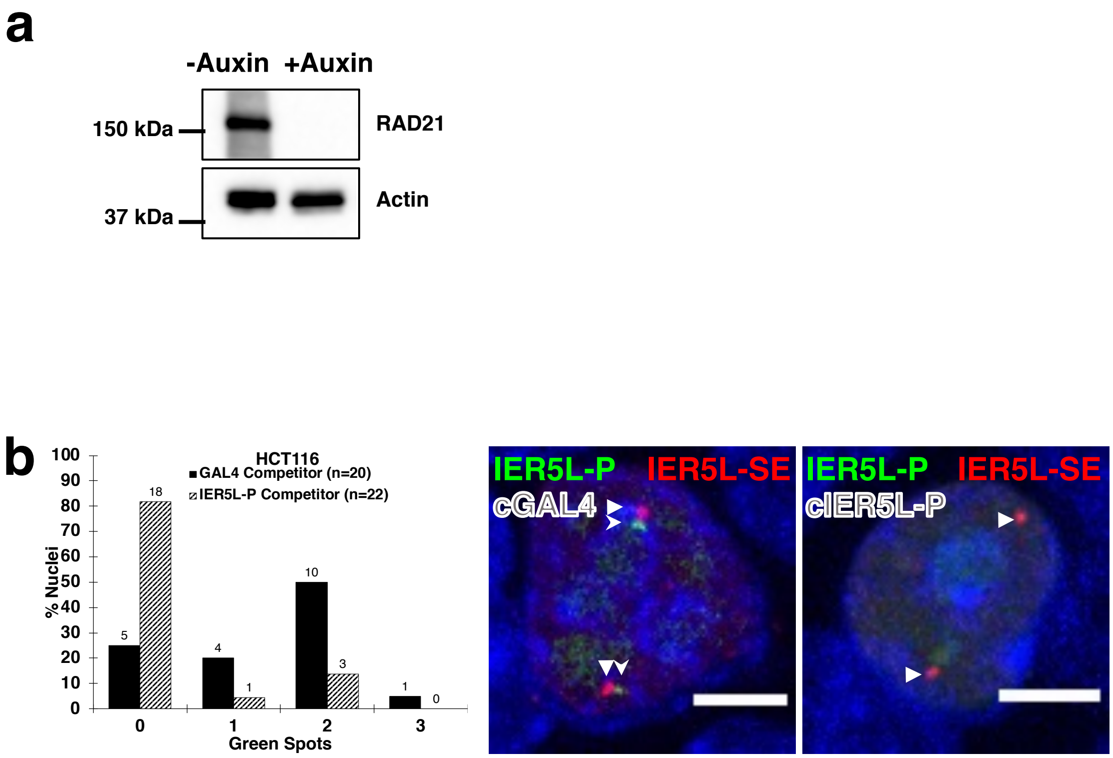

Supplementary Fig 3. (a) RAD21 and actin immunoblot of HCT116/RAD21-mAID cell extracts without and with auxin treatment. Experiment repeated 3 times. **(b)** Competitor experiments for validating *IER5L* P-SE labeling. Column plots show percentages of nuclei with the indicated numbers of Clover (green) spots (counts shown on top of columns) in HCT116 cells co-transfected with Clover-PUFc/gIER5L-P-15xPBSc, PUF9R-iRFP670/gIER5L-SE-15xBPS9R and either a non-competing GAL4 gRNA (black columns) or a gRNA competing with the IER5L-P (patterned columns). Representative images in the non-competed or competed samples (Clover, green spots and stealth arrowheads; iRFP670, red spots and triangle arrowheads) are shown to the right. Scale bars, 5 μm. Experiment repeated 3 times. Source data are provided as a Source Data file.

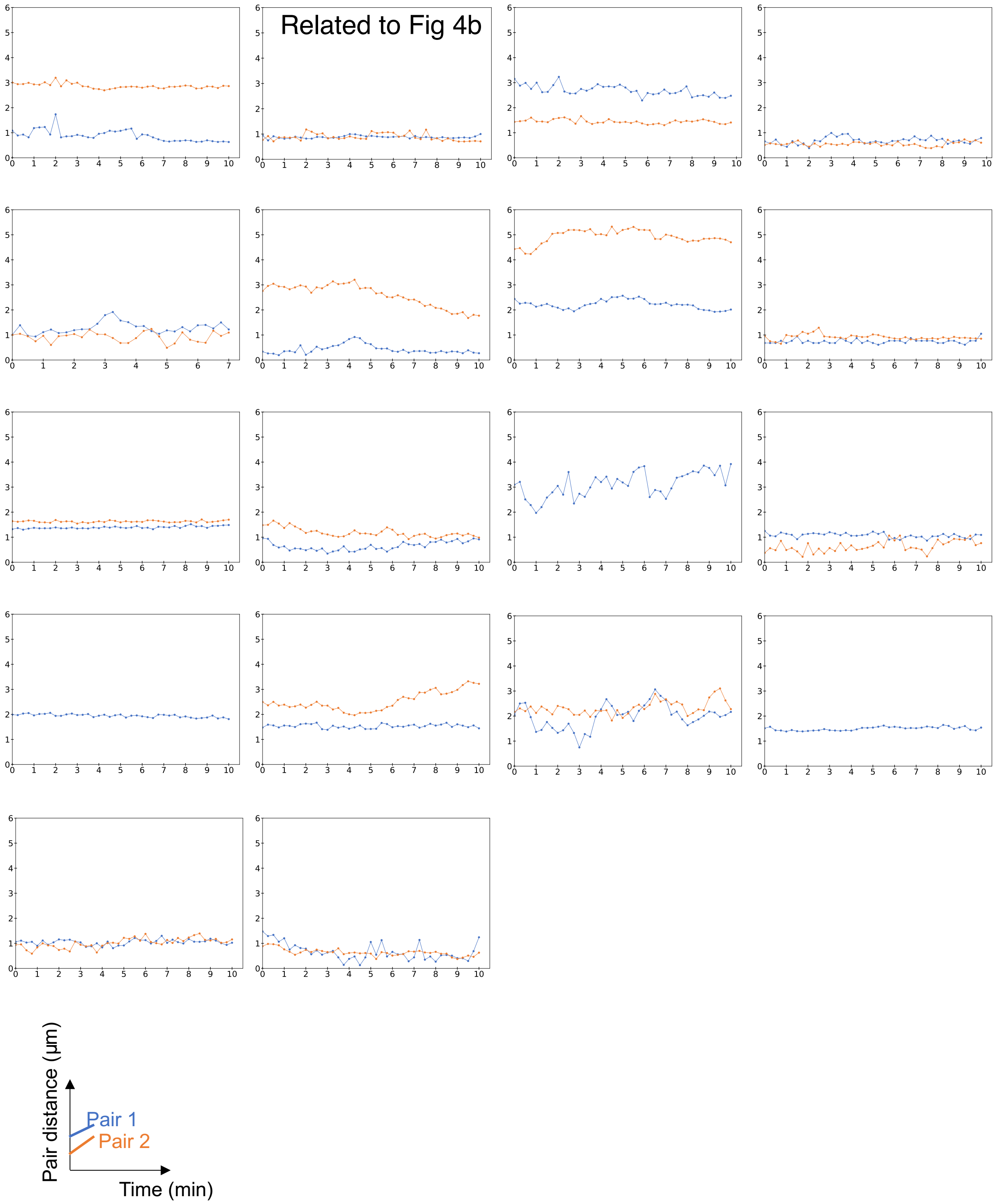

Supplementary Fig. 4. Pairwise 3D distances of Clover and iRFP670 foci labeling the *IER5L*-P locus and *IER5L*-SE locus in 18 nuclei (33 pairs) of untreated HCT116/RAD21-mAID cells over time. Source data are provided as a Source Data file.

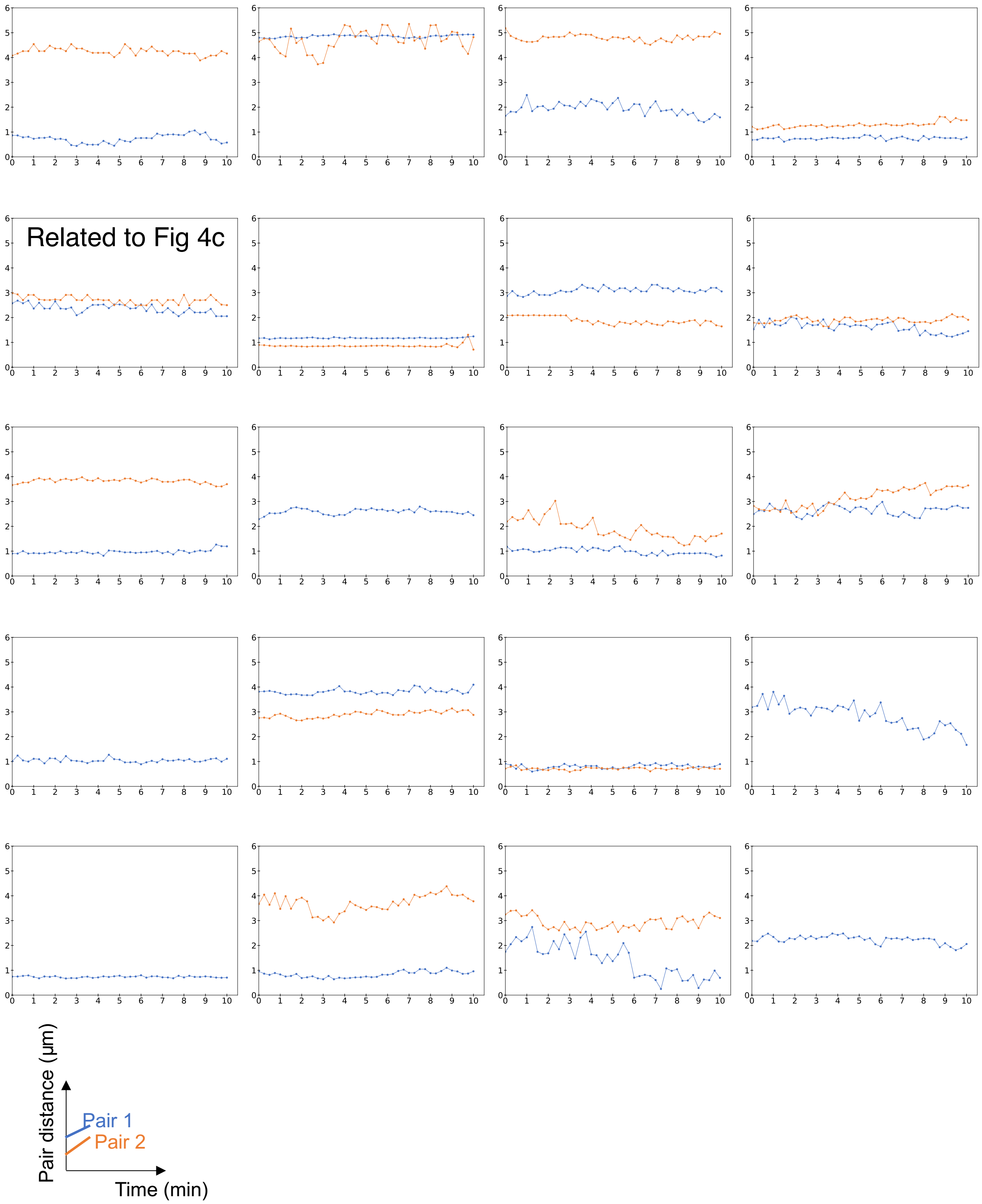

Supplementary Fig. 5. Pairwise 3D distances of Clover and iRFP670 foci labeling the *IER5L*-P locus and *IER5L*-SE locus in 20 nuclei (35 pairs) of auxin-treated HCT116/RAD21-mAID cells over time. Source data are provided as a Source Data file.

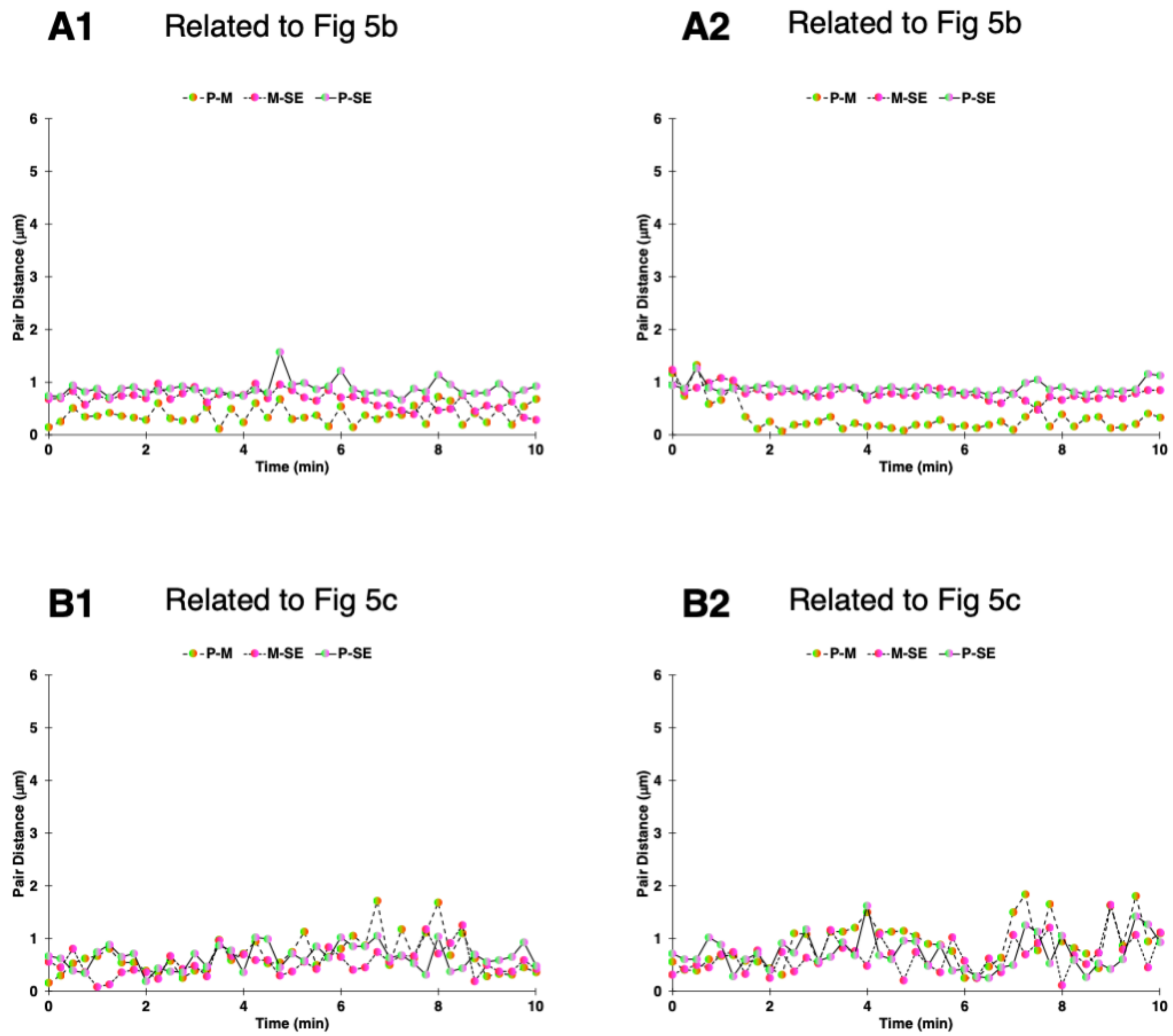

Supplementary Fig. 6. All combinations of pairwise 3D distances of Clover (locus P), iRFP670 (locus M) and mRuby2 (locus SE) spots on the *IER5L* P-SE loop in 10 HCT116/RAD21-mAID nuclei (15 alleles) over time. Source data are provided as a Source Data file.

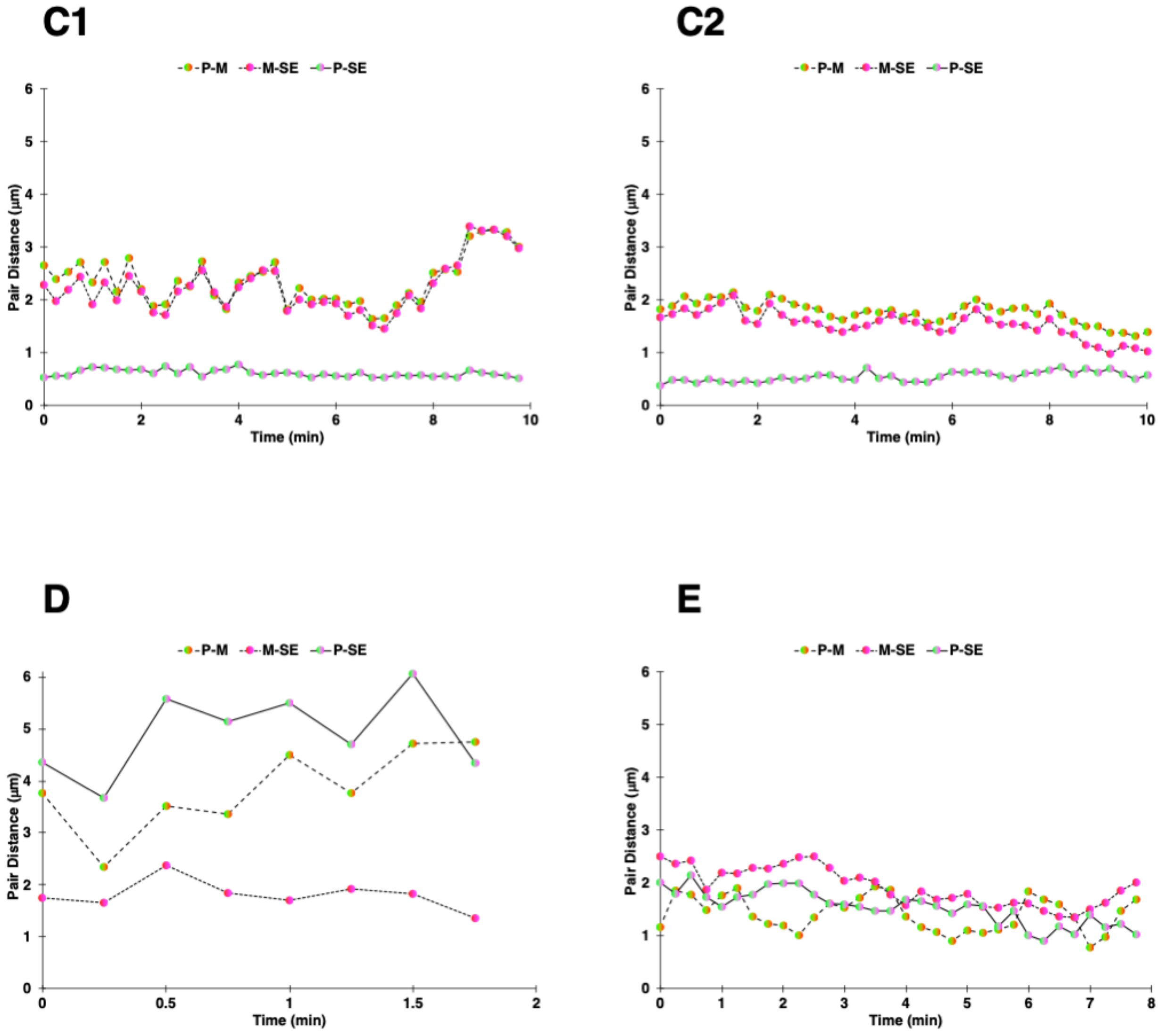

Supplementary Fig. 6 (continued). All combinations of pairwise 3D distances of Clover (locus P), iRFP670 (locus M) and mRuby2 (locus SE) spots on the *IER5L* P-SE loop in 10 HCT116/RAD21-mAID nuclei (15 alleles) over time. Source data are provided as a Source Data file.

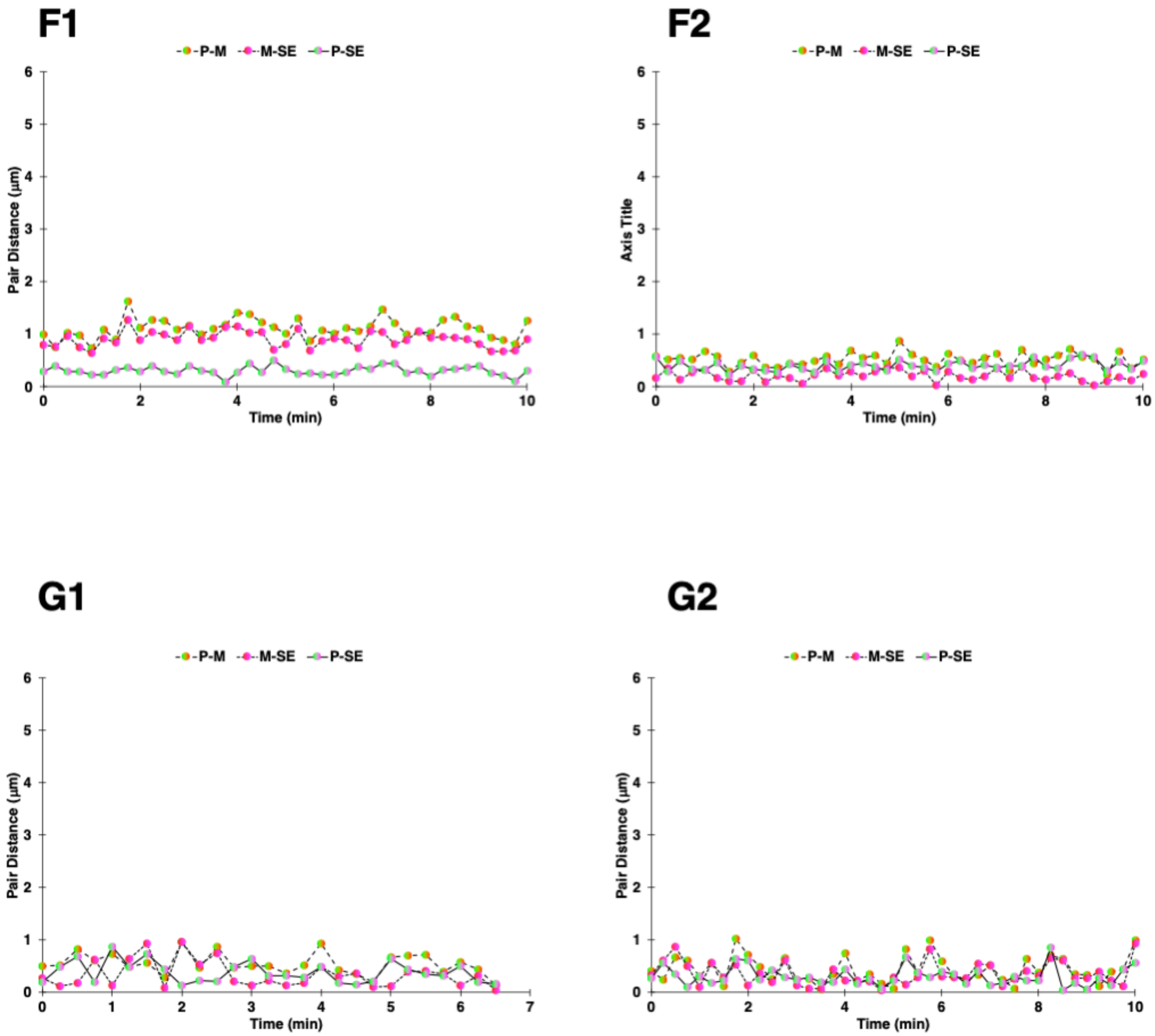

Supplementary Fig. 6 (continued). All combinations of pairwise 3D distances of Clover (locus P), iRFP670 (locus M) and mRuby2 (locus SE) spots on the *IER5L* P-SE loop in 10 HCT116/RAD21-mAID nuclei (15 alleles) over time. Source data are provided as a Source Data file.

H

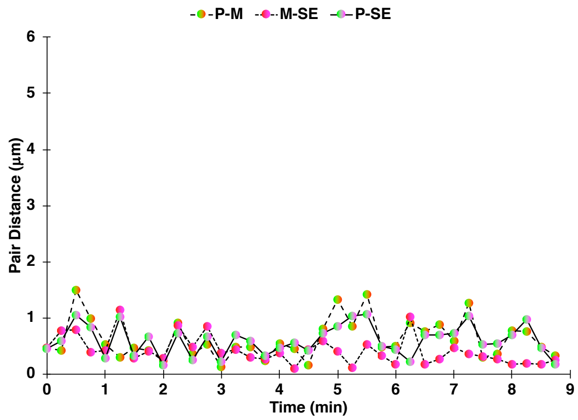

I

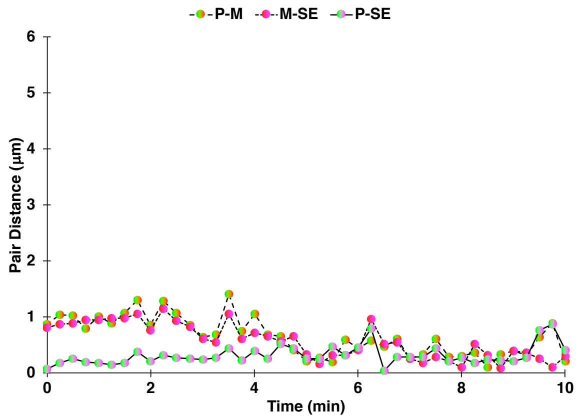

J

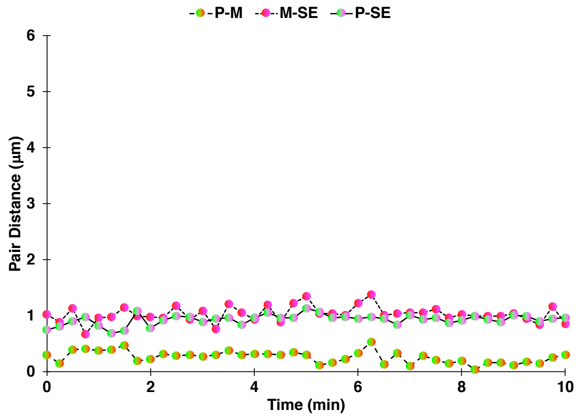

Supplementary Fig. 6 (continued). All combinations of pairwise 3D distances of Clover (locus P), iRFP670 (locus M) and mRuby2 (locus SE) spots on the *IER5L* P-SE loop in 10 HCT116/RAD21-mAID nuclei (15 alleles) over time. Source data are provided as a Source Data file.
