# Supplementary material for: CRISPR-mediated multiplexed live cell imaging of nonrepetitive genomic loci with one guide RNA per locus
Source: Nat Commun. 2022 Apr 6;13:1871. doi: 10.1038/s41467-022-29343-z (PMC8987088; doi:10.1038/s41467-022-29343-z)
Supplement: Supplementary file 2 — Reporting Summary [file 41467_2022_29343_MOESM2_ESM.pdf]

nature research

Corresponding author(s): Albert W. Cheng, Jacqueline J. Zhu

Last updated by author(s): Mar 2, 2022

Reporting Summary

Nature Research wishes to improve the reproducibility of the work that we publish. This form provides structure for consistency and transparency in reporting. For further information on Nature Research policies, see Authors & Referees and the Editorial Policy Checklist .

Statistics

For all statistical analyses, confirm that the following items are present in the figure legend, table legend, main text, or Methods section.

n/a	Confirmed	
☐	☒	The exact sample size (*n*) for each experimental group/condition, given as a discrete number and unit of measurement
☒	☐	A statement on whether measurements were taken from distinct samples or whether the same sample was measured repeatedly
☐	☒	The statistical test(s) used AND whether they are one- or two-sided<br>*Only common tests should be described solely by name; describe more complex techniques in the Methods section.*
☒	☐	A description of all covariates tested
☒	☐	A description of any assumptions or corrections, such as tests of normality and adjustment for multiple comparisons
☐	☒	A full description of the statistical parameters including central tendency (e.g. means) or other basic estimates (e.g. regression coefficient) AND variation (e.g. standard deviation) or associated estimates of uncertainty (e.g. confidence intervals)
☐	☒	For null hypothesis testing, the test statistic (e.g. *F*, *t*, *r*) with confidence intervals, effect sizes, degrees of freedom and *P* value noted<br>*Give P values as exact values whenever suitable.*
☒	☐	For Bayesian analysis, information on the choice of priors and Markov chain Monte Carlo settings
☒	☐	For hierarchical and complex designs, identification of the appropriate level for tests and full reporting of outcomes
☒	☐	Estimates of effect sizes (e.g. Cohen's *d*, Pearson's *r*), indicating how they were calculated

Our web collection on statistics for biologists contains articles on many of the points above.

Software and code

Policy information about availability of computer code

Data collection	MUC4 guide sequence filtered for specificity using crispor.tefor.net.<br><br>Fastq files of ARPE-19 RAD21 ChIA-PET experiment (ENCSR110JOO) were downloaded from ENCODE database, and processed by ChIA-PET2. Interactions were ranked by PET count. Top-ranked interactions with different PET distances were selected. To avoid interference of CTCF binding, design regions were selected by shifting external to PET regions. JACKIE was then used to identify unique 1-copy CRISPR sites in the hg38 genome overlapping design regions of selected loops and further filtered for specificity by Cas-OFFinder. ChIA-PET loops are displayed on WashU EpiGenome Browser (https://epigenomegateway.wustl.edu/).<br><br>Images were acquired with Fusion software (version 2.0.0.15), and processed using Fusion's ClearView-GPU deconvolution with the Robust (Iterative) algorithm (pre-sharpening 0, 5 iterations, and de-noising filter size 0.1).<br><br>Genome tracks were visualized by UCSC Genome Browser. Hi-C maps were generated using FAN-C.
Data analysis	To measure two targeted genomic loci distance in time-lapse images, Fiji image analysis software (ImageJ version 2.1.0/1.53c) was used. Z-series acquired at 0.32 µm step size for 40x objective, and 0.16-0.2 µm step size for 63x objective was used. If the nucleus drifted over time, the Correct 3D Drift plugin was used. For segmenting and tracking spots, the TrackMate plugin was used. Blob diameter was set at 2.0 µm. For each channel, threshold was set to include two spots with the maximum intensity in the 3D volume of the nucleus. Simple LAP Tracker used 2 µm linking max distance, 2 µm gap closing max distance, and 2 gap closing max frame gap. Analysis produced "Spots in track statistics" file which was used to run python script to calculate 3D distances between spots and generate 3D tracks.

For manuscripts utilizing custom algorithms or software that are central to the research but not yet described in published literature, software must be made available to editors/reviewers. We strongly encourage code deposition in a community repository (e.g. GitHub). See the Nature Research guidelines for submitting code & software for further information.

Data

Policy information about availability of data

All manuscripts must include a data availability statement. This statement should provide the following information, where applicable:

- Accession codes, unique identifiers, or web links for publicly available datasets
- A list of figures that have associated raw data
- A description of any restrictions on data availability

All data is available in the main text or the supplementary materials. Source data are provided with this paper. Plasmids are available on Addgene (see Supplementary Table 2 for plasmid listing with links). Raw images[44] are deposited on Dryad repository at https://doi.org/10.5061/dryad.cjsxksn5v. Previously released ARPE-19 ChIA-PET data are available at ENCODE database with accession number ENCSR110JOO [https://www.encodeproject.org/experiments/ENCSR110JOO/]. Previously published HCT116/RAD21-mAID HiC datasets[29] are available at GEO database with accession number GSE104334 [https://www.ncbi.nlm.nih.gov/geo/query/acc.cgi?acc=GSE104334]

Field-specific reporting

Please select the one below that is the best fit for your research. If you are not sure, read the appropriate sections before making your selection.

☒ Life sciences ☐ Behavioural & social sciences ☐ Ecological, evolutionary & environmental sciences

For a reference copy of the document with all sections, see nature.com/documents/nr-reporting-summary-flat.pdf

Life sciences study design

All studies must disclose on these points even when the disclosure is negative.

Sample size	All sample sizes provided in figure legends. No sample size calculation was performed.<br>Sample sizes were chosen as customary in the field and were sufficient to obtain statistical significance.
Data exclusions	No data excluded
Replication	Imaging experiments were repeated 3-10 times, as specified in figure legends. All attempts at replication were successful.
Randomization	Samples were not allocated into groups, thus randomization is not relevant for this study.
Blinding	Samples were not allocated into groups, thus blinding is not relevant for this study.

Reporting for specific materials, systems and methods

We require information from authors about some types of materials, experimental systems and methods used in many studies. Here, indicate whether each material, system or method listed is relevant to your study. If you are not sure if a list item applies to your research, read the appropriate section before selecting a response.

Materials & experimental systems

n/a	Involved in the study	
☒	☐	Antibodies
☐	☒	Eukaryotic cell lines
☒	☐	Palaeontology
☒	☐	Animals and other organisms
☒	☐	Human research participants
☒	☐	Clinical data

Methods

n/a	Involved in the study	
☒	☐	ChIP-seq
☒	☐	Flow cytometry
☒	☐	MRI-based neuroimaging

Eukaryotic cell lines

Policy information about cell lines

Cell line source(s)	U2OS and ARPE-19 are from ATCC; HAP1 from Horizon Discovery; HCT116/RAD21-mAID from Kanemaki lab
Authentication	None of the cell lines used were authenticated.
Mycoplasma contamination	Not tested
Commonly misidentified lines<br>(See ICLAC register)	No commonly misidentified cell lines were used in the study.
