# Supplementary material for: CRISPR-mediated multiplexed live cell imaging of nonrepetitive genomic loci with one guide RNA per locus
Source: Nat Commun. 2022 Apr 6;13:1871. doi: 10.1038/s41467-022-29343-z (PMC8987088; doi:10.1038/s41467-022-29343-z)
Supplement: Supplementary file 4 — Description of Additional Supplementary Files [file 41467_2022_29343_MOESM4_ESM.pdf]

Title: Supplementary Video 1

Description: Time-lapse video of a representative ARPE-19 nucleus visualized by Casilio labeling the *MASP1-BCL6* loop 5' and 3' anchors with Clover (green) and iRFP670 (red), respectively. Top panels, from left to right: Clover (green), iRFP670 (red), merged (scale bar, 5 μm), blue and orange panels correspond to boxed areas of the merged image (scale bars, 1 μm). Lower panel shows the pairwise distance (y-axis, μm) with line colors corresponding to the boxed pairs (x-axis, minutes). Related to Fig. 3b.

Title: Supplementary Video 2

Description: Time-lapse video of representative untreated HCT116/RAD21-mAID nuclei visualized by Casilio labeling the *IER5L* promoter with Clover (green) and *IER5L* super-enhancer with iRFP670 (red), respectively. Top panels, from left to right: Clover (green), iRFP670 (red), merged (scale bar, 5 μm), blue and orange panels correspond to boxed areas of the merged image (scale bars, 1 μm). Lower panel shows the pairwise distance (y-axis, μm) with line colors corresponding to the boxed pairs (x-axis, minutes). Related to Fig. 4b.

Title: Supplementary Video 3

Description: Time-lapse video of representative auxin-treated HCT116/RAD21-mAID nuclei visualized by Casilio labeling the *IER5L* promoter with Clover (green) and *IER5L* super-enhancer with iRFP670 (red), respectively. Top panels, from left to right: Clover (green), iRFP670 (red), merged (scale bar, 5 μm), blue and orange panels correspond to boxed areas of the merged image (scale bars, 1 μm). Lower panel shows the pairwise distance (y-axis, μm) with line colors corresponding to the boxed pairs (x-axis, minutes). Related to Fig. 4c.

Title: Supplementary Video 4

Description: Time-lapse video of representative untreated HCT116/RAD21-mAID nuclei visualized by Casilio labeling a RAD21-independent loop. Top panels, from left to right: Clover (green), iRFP670 (red), merged (scale bar, 5 μm), blue and orange panels correspond to boxed areas of the merged image (scale bars, 1 μm). Lower panel shows the pairwise distance (y-axis, μm) with line colors corresponding to the boxed pairs (x-axis, minutes). Related to Fig. 4f.

Title: Supplementary Video 5

Description: Time-lapse video of representative auxin-treated HCT116/RAD21-mAID nuclei visualized by Casilio labeling a RAD21-independent loop. Top panels, from left to right: Clover (green), iRFP670 (red), merged (scale bar, 5 μm), blue, orange and yellow panels correspond to boxed areas of the merged image (scale bars, 1 μm). Lower panel shows the pairwise distance (y-axis, μm) with line colors corresponding to the boxed pairs (x-axis, minutes). Related to Fig. 4g.

Title: Supplementary Videos 6

Description: PISCES – 3-color 3-point live-cell time-lapse imaging of the *IER5L* promoter-super enhancer loop with promoter, mid-point, super-enhancer labeled by Clover (green, first left panel), iRFP670 (red, second panel), and mRuby2 (magenta, third panel). Right panel shows merged image. Scale bars, 5 μm. Related to Fig. 5b.

Title: Supplementary Videos 7

Description: PISCES – 3-color 3-point live-cell time-lapse imaging of the *IER5L* promoter-super enhancer loop with promoter, mid-point, super-enhancer labeled by Clover (green, first left panel), iRFP670 (red, second panel), and mRuby2 (magenta, third panel). Right panel shows merged image. Scale bars, 5 μm. Related to Fig. 5c.
